# Effect of mineralogical composition on the compressive strength and microstructure of metakaolin geopolymer

**DOI:** 10.1038/s41598-026-49264-x

**Published:** 2026-05-04

**Authors:** H. Abdeen, Alaa Mohsen, AbdelMonem Soltan, Mohamed Kohail

**Affiliations:** 1 Faculty of Engineering, Ain Shams University, Cairo, Egypt; 2 Geology Department, Faculty of Science, Ain Shams University, Cairo, 11566 Egypt

**Keywords:** Geopolymer, Metakaolin, Pozzolanic activity, Mineralogical composition, Compressive strength, Engineering, Environmental sciences, Materials science, Solid Earth sciences

## Abstract

The objective of this work is to investigate the impact of mineralogical composition (SiO_2_ and Al_2_O_3_ content) on the properties of metakaolin-based geopolymers, including compressive strength and microstructure. Two kaolin samples with different mineralogical compositions were used: one has higher Al_2_O_3_ and lower SiO_2_ content (coded as kH), compared to the second one, which is coded as kL. Post calcination at 700 °C for 1 h, the pozzolanic activity of both metakaolin samples was measured by the Chapelle test. The metakaolin samples (MkH and MkL) were activated with NaOH and Na_2_SiO_3_ to produce geopolymers. Four mixes from each metakaolin sample were prepared based on varying SiO_2_/Al_2_O_3_ ratios at 3.1, 3.3, 3.5 and 3.7. Initial and final setting time and compressive strength were evaluated to assess the quality of the resulting geopolymers. The phase composition of the geopolymers was analyzed using X-ray diffraction (XRD) and thermogravimetric analysis (TGA/DTG), as well as microstructure using scanning electron microscopy (SEM) and the Brunauer–Emmett–Teller/Barrett–Joyner–Halenda models (BET/BJH). The presence of Al_2_O_3_ in kaolin has significantly improved the metakaolin reactivity. MkH and MkL have pozzolanicity of 1006 and 767 mg CaO/g Mk, respectively. The MkH with a low SiO_2_ content requires high Na_2_SiO_3_ to produce a geopolymer with the same SiO_2_/Al_2_O_3_ ratio as the MkL-based geopolymer. The highest compressive strength was 64.24 and 18.6 MPa at a SiO_2_/Al_2_O_3_ molar ratio of 3.5 for MkH- and MkL-based geopolymers, respectively. This suggests that adding SiO_2_ externally with Na_2_SiO_3_ in the geopolymer would be more effective than relying on its presence in the original mineral makeup of the raw material metakaolin. The soluble active SiO_2_ included in the matrix by Na_2_SiO_3_ promotes the rapid formation of a more densely packed network structure.

## Introduction

With population growth, the world is increasingly focusing on developing sustainable urban communities by using alternative building materials instead of cement due to its environmental issues^1^. Cement production depends on natural raw materials in the manufacturing process and consumes a huge amount of energy^2^. Furthermore, it is a major contributor to global warming, as every ton of cement produces approximately 0.8 tons of CO_2_. One practical approach is to use blended cement by partially replacing cement with industrial or agricultural wastes^3^. Commonly, these wastes are characterized by a high content of SiO_2_ and Al_2_O_3_, such as silica fume, fly ash, dealuminated metakaolin, rice husk biochar and granite powder. They can enhance the compressive strength by densifying the cement matrix and promoting the formation of additional hydration products. For instance, adding dealuminated metakaolin to cement can improve compressive strength by refining the microstructure and lowering the Ca/Si and Ca/Al ratios compared to pure cement, which indicates the formation of longer/polymerized calcium silicate hydrate (C–S–H) and calcium aluminate hydrate (C–A–H)^1^. The high SiO_2_ and Al_2_O_3_ content in the rice husk biochar increases the amount of the formed strength-giving phases^4^. Also, the granite powder enhanced the compressive strength through the combined pozzolanic and filler effect^5^.

Recently, sustainable building materials have been developed alongside the emergence of cement‑free binders, such as geopolymer^6^. Several studies reported that relying on geopolymer instead of cement will conserve a massive amount of natural materials and reduce about 60–80% of CO_2_^7^. Alongside the environmental friendliness of geopolymers, they have superior mechanical strength and high durability^8,9^. They are produced through the alkali-activation of aluminosilicate materials, such as metakaolin, resulting in a three-dimensional network of polymer chains, offering significant benefits for construction uses^10^. A key factor influencing the properties of geopolymer materials is the mineralogical composition of aluminosilicate precursors. Silica plays a vital role in geopolymerization by reacting with aluminates to form polymer networks. Different sources of silica, such as metakaolin, rice husk ash, fly ash, silica fume, and sodium silicate, have been studied for their suitability in geopolymer synthesis^11–15^. Additionally, comparisons have been made between metakaolin types sourced from different regions due to their distinct chemical compositions and properties^16–19^. However, the impact of different silica sources on the quality of metakaolin geopolymers is still an area of ongoing research.

The compressive strength and microstructural development of metakaolin geopolymers mainly depend on the amount of active silica available in metakaolin or added sodium silicate, which was expressed as a mass ratio of Na_2_SiO_3_/NaOH^12,20–27^ or SiO_2_/Al_2_O_3_ molar ratio^26,28–31^ in the studies. Different researchers have reported the optimal mass ratio of Na_2_SiO_3_ to NaOH to be approximately 1.0–2.5^12,20,21,23,24^. Other researchers have reported different views of the SiO_2_/Al_2_O_3_ ratio^24,31–36^. Ghanbari et al.^32^ reported that the optimum molar ratio of SiO_2_/Al_2_O_3_ is 2.9, which was confirmed by Alekseev et al.^24^, as they also proved that optimum molar ratio falls between 2.5 and 3.0. This is different from the results of Silva et al.^35^ and Juengsuwattananon et al.^31^, which suggested using SiO_2_/Al_2_O_3_ in the range of 3.4–3.8. Yunsheng et al.^36^ concluded that the highest compressive strength of 34.9 MPa was achieved at a SiO_2_/Al_2_O_3_ ratio of 5.5.

For a more comprehensive overview of previous studies that employed different types of metakaolin as an aluminosilicate source and sodium silicate as an additional silica source for geopolymer production, many of these studies are summarized in Table 1. It is observed that the high Al_2_O_3_ wt.% in metakaolin results in the production of higher quality geopolymers with superior compressive strength. Albidah et al.^20^ used Saudi Arabia metakaolin containing 42.63 wt.% Al_2_O_3_ to produce a geopolymer with a compressive strength of 58.5 MPa. Similarly, Riahi et al.^37^. employed Iranian metakaolin with 45 wt.% Al_2_O_3_, resulting in a geopolymer with a strength of 60.6 MPa. Additionally, Mo et al.^38^ used Chinese metakaolin with 43.5 wt.% Al_2_O_3_, achieving a geopolymer with a high compressive strength of 97.95 MPa. While Ozer et al.^39^ utilized Turkish metakaolin containing 41.04 wt.% Al_2_O_3_, resulting in a geopolymer with a compressive strength of 23 MPa. This relatively low strength likely stems from the SiO_2_/Al_2_O_3_ ratio of 4.4 used in the geopolymer, which exceeds the recommended range of 3.0–3.8 found in related studies^29,33,35,40^. When the Al_2_O_3_ wt.% decreased to 38.6 (Jaya et al.^21^) and 35.7 (Silva et al.^35^), the strength also dropped to 32 and 22 MPa, respectively. This provides strong evidence of the correlation between alumina content in metakaolin and the strength achieved by the geopolymer. Although the influence of aluminosilicate chemistry, particularly the SiO_2_/Al_2_O_3_ ratio, and other parameters such as composition/modulus of activator and curing regime on geopolymer properties has been widely reported, fewer studies have systematically focused on the effect of available/reactive Al in metakaolin as a precursor-controlled parameter influenced by kaolin source and calcination conditions. Currently, there is some speculation about the exact role of Al_2_O_3_ content in the properties of geopolymers. One theory states that the Al_2_O_3_ content in metakaolin plays a crucial role in determining these properties. The significance of the high Al_2_O_3_ content in metakaolin becomes evident in improving its reactivity and enabling it to bond with other silicate (SiO_2_) compounds present in metakaolin or added from sodium silicate (Na_2_SiO_3_), thereby producing a geopolymer with high compressive strength^41^. While the other theory suggests that the Al_2_O_3_ content is not only unimportant but also negatively impacts the properties of the geopolymer when it exceeds a specific limit. Silva et al.^35^ reported that the Al_2_O_3_ content in metakaolin decreases SiO_2_/Al_2_O_3_ molar ratios, which promotes the formation of poly(sialate) polymers during geopolymerization. This results in forming a brittle and weak structure with limited hydraulic activity. A low SiO_2_/Al_2_O_3_ ratio creates a less cross-linked, two-dimensional network, compared to the stronger, three-dimensional network that provides better strength at higher molar ratios. Ababneh et al.^42^ concluded that kaolin samples with higher Si/Al ratios are more likely to produce alkali-activated binders with greater compressive strength. Higher (Fe + Al) contents in calcined kaolin were found to decrease the compressive strength of the alkali-activated binder. It has been reported that Fe^3^^+^ can replace Al^3+^ within the aluminosilicate structure, potentially lowering the Si/Al ratio and reducing compressive strength. Furthermore, a high Fe_2_O_3_ content was observed to delay the geopolymerization reaction by forming an iron silicate-based binder. Other studies link the amorphous maturity of metakaolin to its degree of pozzolanicity and subsequently to the compressive strength of the produced geopolymer. Kakali et al.^43^ reported that lower-order kaolinite is more reactive than well-ordered kaolinite.

**Table 1:** Previous literature that adopted metakaolin as a source of alumina silicate and a Na_2_SiO_3_ as an additional silica sources.

Kaolin area	Major oxides of kaolin			Calcination condition	Optimum parameters for prepared geopolymer				Compressive strength (MPa)	References
	SiO_2_	Al_2_O_3_	SiO_2_/Al_2_O_3_		SiO_2_/Al_2_O_3_	Liquid/solid	Na_2_SiO_3_/NaOH	NaOH (molar)		
Saudi Arabia	51	42.63	2.03	750 °C 3h	2.69	0.85	2	14	58.5 (28d)	Albidah et al.^20^
Malaysia	55.7	38.6	2.45	850 °C 6h	2.98	0.8	1.0	10	32 (28d)	Jaya, et al.^21^
Malaysia	54.1	33.0	2.78	800 °C 2h	3.10	1.25	0.2	8	~ 5.5 (7d)	Liew et al.^22^
Nigeria	60.09	33.23	3.07	700 °C 2h	3.1	0.75	0.24	10	11.05 (28d)	Ayeni et al.^12^
Iran	53	45	2.00	–	3.0–3.5	–	–	10	60.6 (7d)	Riahi et al.^37^
China	52.98	43.50	2.07	800 °C 3h	3.3	–	–	–	97.95 (7d)	Mo et al.^38^
Australia	47.3	35.7	2.25	–	3.4–3.8	–	–	–	22 (3d)	Silva et al.^35^
UK	SiO_2_/Al_2_O_3_ = 2.3			–	3.8	–	–	–	~ 82 (7d)	Duxson et al.^29^
Turkey	56.21	41.04	2.32	700 °C 1h	4.4	–	–	8	23 (28d)	Ozer et al.^39^
Brazilian	53.65	31.79	2.86	–	4.49	1.25	2.5	10	39.36 (28d)	da Silva Neto et al.^23^
Morocco	66.47	20.26	5.57	750 °C 3h	6.64	0.67	2.5	14	34.81 (28d)	Aouan et al.^44^
Russia	55.2	43.7	1.26	750 °C 2h	2.5–3.0	0.63	1.0	10	67.0 (7d)	Alekseev et al.^24^

According to the previous literature, this study aims to investigate how mineralogical composition (SiO_2_ and Al_2_O_3_ content) affects the quality and properties of metakaolin-based geopolymers, including compressive strength and microstructure. Understanding the effect of differences in silica sources (metakaolin itself or Na_2_SiO_3_) on geopolymer quality not only helps optimize formulations but also provides valuable insights into the fundamental mechanisms of geopolymerization. To achieve our objectives, a comprehensive experimental campaign will be conducted. Two types of metakaolin will serve as the primary aluminosilicate precursor for geopolymer synthesis. Sodium silicate (Na_2_SiO_3_), another source of silica, will be used to increase and modify the total silica content in the geopolymer mixes. The obtained geopolymer samples will then undergo various characterization techniques, including thermal analysis (TGA and DTG), X-ray diffraction (XRD), scanning electron microscopy (SEM), and Brunauer–Emmett–Teller (BET and BJH) methods. These techniques will offer insights into the crystalline phases, microstructure, and porosity of the geopolymers, enabling a thorough assessment of their quality. The findings of this study will add to the growing knowledge about metakaolin geopolymers and their potential uses. By understanding how different silica sources affect geopolymer quality, researchers and practitioners can make better decisions about material choices and improvements, ultimately creating more sustainable construction materials with adequate compressive strength.

## Materials and experimental program

### Materials

In this study, two different Egyptian kaolinitic clays from Sinai, Egypt, were used to demonstrate how mineralogical composition influences the properties of metakaolin geopolymer. The kaolin samples have varying SiO_2_ and Al_2_O_3_ wt.%; one has low Al_2_O_3_ wt.% and the other has high wt.%, labeled as kL and kH, respectively. The samples were crushed using a jaw crusher and then milled with a ball mill. Thermal analysis (TGA and DTG) of the kaolin samples was performed using a NETZSCH STA 449F5 instrument, operated at a heating rate of 20 °C/min in N_2_ gas, to identify the calcination temperature range for producing metakaolin with high pozzolanicity. The optimal calcination temperature was determined by calculating the dehydroxylation degree (D, %), which is a key indicator of kaolin’s conversion into metakaolin during heating, as shown in Eq. 1^45,46^.

1 $$D=\left(1-\frac{{M}_{max}-M}{{M}_{max}}\right)*100$$

where M is the mass loss % at a specific temperature and Mmax is the total mass loss% resulting from complete dehydroxylation, measured either by chemical analysis as a loss on ignition (L.O.I) or by TGA when the mass loss becomes nearly constant.

For greater accuracy, the optimal calcination temperature and duration were verified using the Chapelle test, according to NF P 18–513^47^. The Kaolin samples were calcined in an electric furnace at the obtained calcination-temperature range for 0.5, 1.0 and 1.5 h. The calcined kaolin samples were subsequently removed from the furnace and allowed to cool naturally in air, as is the common practice in many previous studies performed by Alujas et al.^48^, Ilić et al.^46^, Dhar et al.^49^ and Khaled et al.^50^. The main aims of air cooling are to (i) maintain the amorphous structure of calcined kaolin by preventing the recrystallization of metastable amorphous phases^51^; and (ii) preserve the structural disorder. During the calcination process, the dehydroxylation reaction refers to the removal of the structurally bound water, as well as the transformation of stable octahedral Al to reactive pentahedral and tetrahedral sites^52,53^. Rapid cooling halts further relaxation and leads to cation redistribution; therefore, these defects persist instead of reorganizing into more stable ordered structures^54,55^. Generally, these features are translated into increasing pozzolanic activity^50,56^. In the Chappelle test, the pozzolanicity was measured as a function of the amount of calcium hydroxide (Ca(OH)_2_) consumed by 1 g of metakaolin when mixed with 2 g of calcium oxide (CaO) and 250 mL of distilled water. The mixture was stirred continuously while heating at 90 °C for 24 h, then cooled to room temperature. Free Ca(OH)_2_ was quantified using sucrose and acid titration. Fresh sucrose solution (250 mL, made by dissolving 60 g of sugar in 250 mL of distilled water) was added to the previous mixture and stirred for 15 min. After that, the final mixture was filtered, and 25 mL of the filtrate was titrated with 0.1N HCl using a phenolphthalein indicator. Finally, the pozzolanic activity was determined using Eq. 2.

2 $$P=2\times \frac{({V}_{1}-{V}_{2})}{{V}_{1}}\times \frac{74}{56}\times 1000$$

where P is the pozzolanicity of Mk (mg calcium oxide (CaO) consumed/g Mk); V_1_ and V_2_ are the volumes of 0.1 N HCl (ml) required to titrate 25 mL of the resulting filtered solution without metakaolin (blank) and with metakaolin, respectively; 74 and 56 are the molecular weights of calcium hydroxide (Ca(OH)_2_) and calcium oxide (CaO), respectively.

The kaolin and metakaolin were analyzed using different techniques to show the impact of the calcination process. The chemical composition was investigated using an X-ray fluorescence spectrometer (XRF: Xios, stylePW-1400). The phase composition was studied using X-ray diffraction (XRD: Philips model Xpert-2000 diffractometer), carried out with 40 kV power, an X-ray with λ = 1.542 Å CuKα radiation, scanning from ranges 2θ = 2–60° and scanning speed of 0.02°/sec. Also, the morphology was examined using a scanning electron microscope (SEM: Thermo-Scientific/Quattro-S).

The alkaline activating solution used to prepare metakaolin-based geopolymer was prepared from sodium hydroxide (NaOH) and sodium silicate (Na_2_SiO_3_) purchased from El-Gomhoria Chemical Company. The NaOH pellet used has a purity of 98% and the Na_2_SiO_3_ solution has a modulus ratio (SiO_2_/Na_2_O) = 2.6, which contains 30.12% SiO_2_, 11.42% Na_2_O and 58.46% H_2_O.

### Experimental program

Firstly, eight geopolymeric mixes divided into four groups were prepared to assess the effects of the mineralogical composition of metakaolin on the properties of geopolymers. The metakaolin with the highest pozzolanic activity for both types was selected for preparing the geopolymer. Two mixes in the one group were prepared using different types of metakaolin (MkL and MkH). In each group, the SiO_2_/Al_2_O_3_ was maintained at 3.10, 3.30, 3.50 and 3.70 for the 1st, 2nd, 3rd and 4th groups, respectively. These ratios were selected based on previous studies by Silva et al.^35^, Rahier et al.^45^ and Duxson et al.^29^. Moreover, within groups, SiO_2_/Al_2_O_3_, Al_2_O_3_/Na_2_O and H_2_O/Na_2_O were maintained at specific values. The H_2_O/Na_2_O ratio for all mixes was constant at 13.00. While the Al_2_O_3_/Na_2_O ratio was 1.20 for the first three groups, it was lowered to 1.00 for the 4th group because the sample with high SiO_2_/Al_2_O_3_ (3.7) could not be mixed at Al_2_O_3_/Na_2_O = 1.20. These mix-designs will be described below in Table 3. Generally, these ratios were achieved by varying the composition of alkaline activating solution (Na_2_SiO_3_/NaOH mass ratio, NaOH concentrations, and liquid/solid ratio (L/S) mass ratios), as reported by Albidah et al.^20^, Huang and Wang^57^, Juengsuwattananon et al.^31^ and Rowles and O’Connor^30^. Due to the difference in the chemical composition (SiO_2_ and Al_2_O_3_ wt.%) of the two metakaolin samples (MkL and MkH), maintaining the target SiO_2_/Al_2_O_3_ ratios in each group requires adjusting the mass of Na_2_SiO_3_ solution added to the mixes. At the same time, because Na_2_SiO_3_ contributes both SiO_2_ and Na_2_O, increasing or decreasing its portions alters the total Na_2_O wt.% in the mixes. Accordingly, to keep the Al_2_O_3_/Na_2_O ratio at 1.20 or 1.00 and the H_2_O/Na_2_O ratio at 13.0 across all mixes, the molarity of the NaOH solution must be changed to compensate for the changes in Na_2_O contributed by Na_2_SiO_3_.

The geopolymeric pastes were prepared by mixing metakaolin with alkaline activating solutions based on each mix-design in a Hobart mixer. After that, they were cast into 1-inch cubic molds. The molded specimens were kept at room temperature (20 ± 5 °C) and 95 ± 5% relative humidity until they were demolded to allow setting of fresh paste, gaining sufficient early strength, minimizing the alkaline solution evaporation, which can induce microcracks or shrinkage^58^. Also, it has been previously proven that extended pre-curing at room temperature before applying heat helps improve strength development^59,60^. Subsequently, the specimens were cured in an oven at 60 °C for 24 h to accelerate the geopolymerization process (dissolution/condensation/polymerization) and boost the early strength development^61^. Particularly, heat-curing at 60 °C for 24 h is commonly reported as an effective condition for metakaolin-based geopolymers^62,63^. After heat curing, all geopolymer samples were kept at room temperature (20 ± 5 °C) and 95 ± 5% relative humidity again until the testing day (28-days). This allows ongoing polycondensation and structural development, while avoiding pore coarsening and strength loss associated with extended heat curing^64^. A similar curing protocol was mentioned by previous studies, such as that performed by Khaled et al.^50^, Ozer and Soyer-Uzun^39^ and Aouan et al.^44^. They placed the molds at 60–70 °C for 24 h immediately after casting, then cured the specimens at room temperature. In this study, the molds were initially cured at room temperature, then heat cured to take advantage of the benefits of pre-curing at room temperature as described above.

The initial and final setting of the prepared metakaolin geopolymers were measured using Vicat apparatus according to ASTM C191-19^65^. The average compressive strength of three replicate samples from each mix was measured with a compression testing machine (maximum load 250 kN). The phase composition of hardened specimens was analyzed using XRD and TGA/DTG. The microstructure was monitored using SEM. The texture parameters were analyzed using pore-size distribution and surface-area analyzer (MICROTRAC-BELSORP/MINI-X). based on the Brunauer–Emmett–Teller model (BET) and the Barrett–Joyner–Halenda model (BJH).

## Results and discussion

### Optimization of kaolin into metakaolin

#### Thermal analysis

**Fig. 1:**
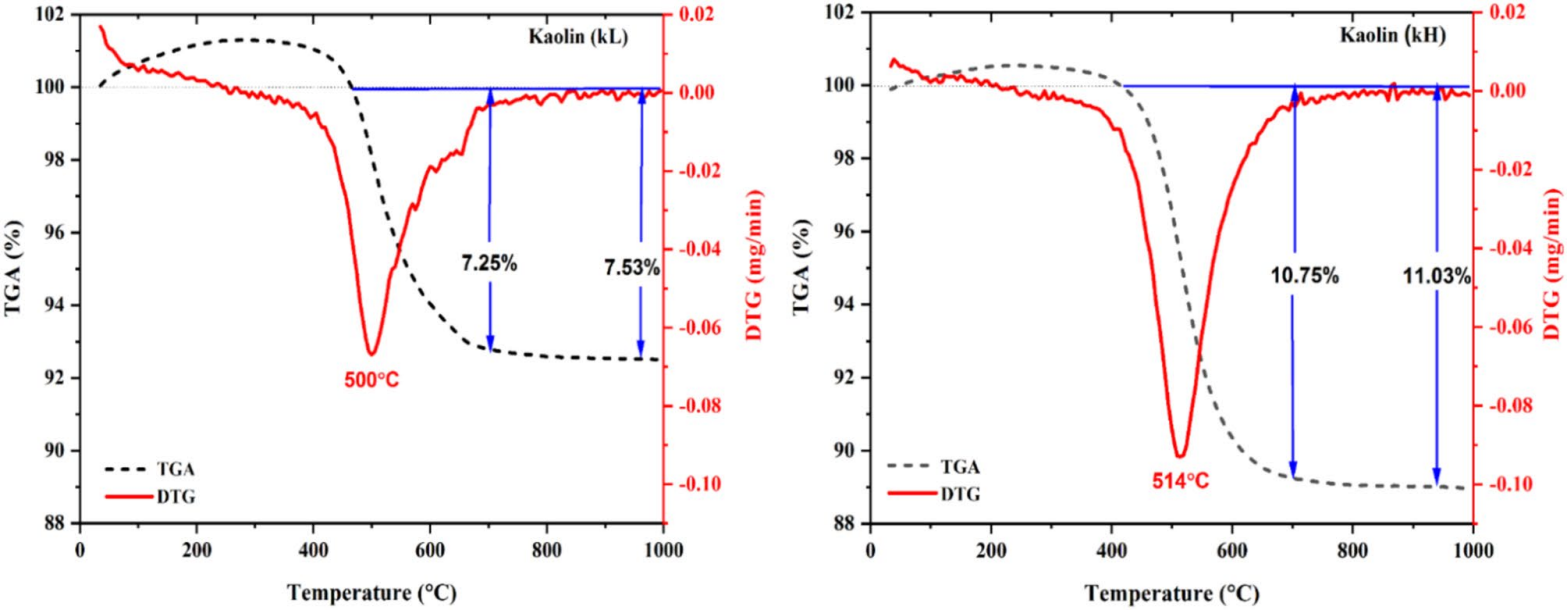
TGA-DTG diagrams of kaolin samples.

To determine the optimal calcination temperature range for the two types of kaolin (kL and kH), their thermal behaviors were analyzed using TGA/DTG, as shown in Fig. 1. The TGA analysis shows mass loss% in kL and kH, reaching 7.53 and 11.03%, respectively. These values will be considered as Mmax, which is used in calculating the degree of dehydroxylation. The DTG profile of kL displays an endothermic peak associated with dehydroxylation of kaolin, occurring alongside the formation of metakaolin, within the temperature range of 450–600 °C, centered at 500 °C. For kH, the endothermic peak appears between 440 and 590 °C, with a center around 514 °C. The high mass loss% and high dehydroxylation temperature of the kH sample compared to the kL suggest that the amount of kaolinite in kH is greater than in kL. This will be verified below by XRD analysis (Fig. 4).

**Fig. 2:**
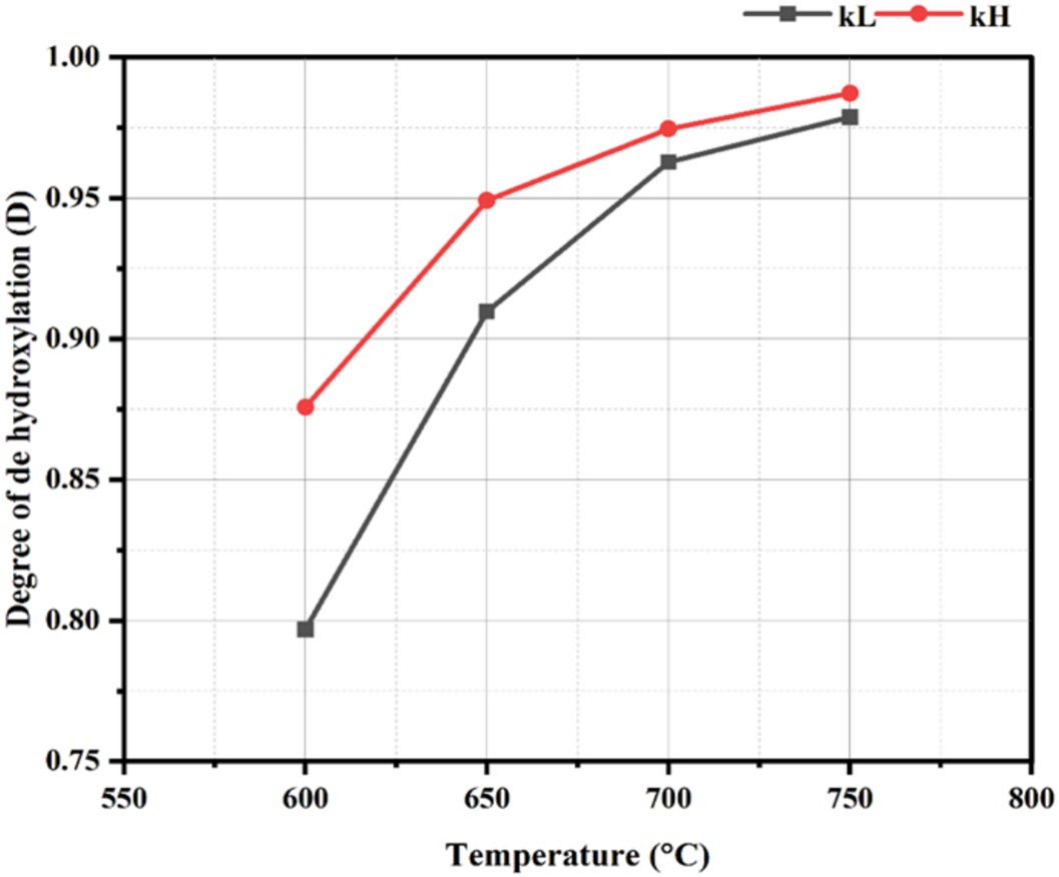
Degree of de-hydroxylation versus calcination temperatures for kaolin samples.

To determine the optimal calcination temperature for both kaolin samples, the degree of dehydroxylation (D) was calculated as shown in Fig. 2. It can be detected that the mass loss% increases at calcination temperatures of 600–700 °C, while higher heating has a negligible impact. The D values of kL and kH samples at 700 °C are 0.963 and 0.975, respectively, exceeding the value that refers to achieving the complete de-hydroxylation, which is 0.95^66^.

#### Chapelle test

Generally, the Chapelle test is used to measure the reactivity of calcined clays through lime fixation and is standard for assessing the pozzolanicity of lime-based systems. However, it is not a definitive indicator for highly alkaline geopolymer systems, where portlandite is absent, as Stefanini et al.^67^. Despite that, they used this test in evaluating the pozzolanicity of calcined waste clays generated from kaolinite extraction to prepare geopolymeric materials. They reported that the Chapelle test was applied as a potential indicator of calcined clays’ reactivity within the alkaline activator solution, since there is no specific direct method for classifying their reactivity in the geopolymerization process. They based their argument on the fact that reactive Si and Al can interact with the alkaline activator solution, like interaction with portlandite. Furthermore, they suggested that a possible link between the reactivity of calcined clays in the supplementary cementitious materials and geopolymeric systems could exist. Although it is essential to consider several other factors that affect the reaction kinetics of calcined clays in the geopolymerization process, such as their morphology, particle size, mix-design, including the blend with other precursors and type/concentration of alkaline activator. Similarly, many studies performed by Khaled et al.^50^, Ashfaq et al.^68^, Ababneh^42^ and Sayed et al.^69^ have relied on the Chapelle test in the evolution of the pozzolanicity of calcined clay to be employed in the preparation of geopolymer. In this study, the results obtained from the Chapelle test were used to corroborate the calcination screening identified by the degree of dehydroxylation from TGA/DTG analysis and to prove the formation of amorphous and reactive aluminosilicate precursors. The data from the Chapelle test will also be verified by monitoring changes in the kaolinite phase of the kaolin samples before and after calcination, through studying the phase composition using XRD, as will be discussed below.

**Fig. 3:**
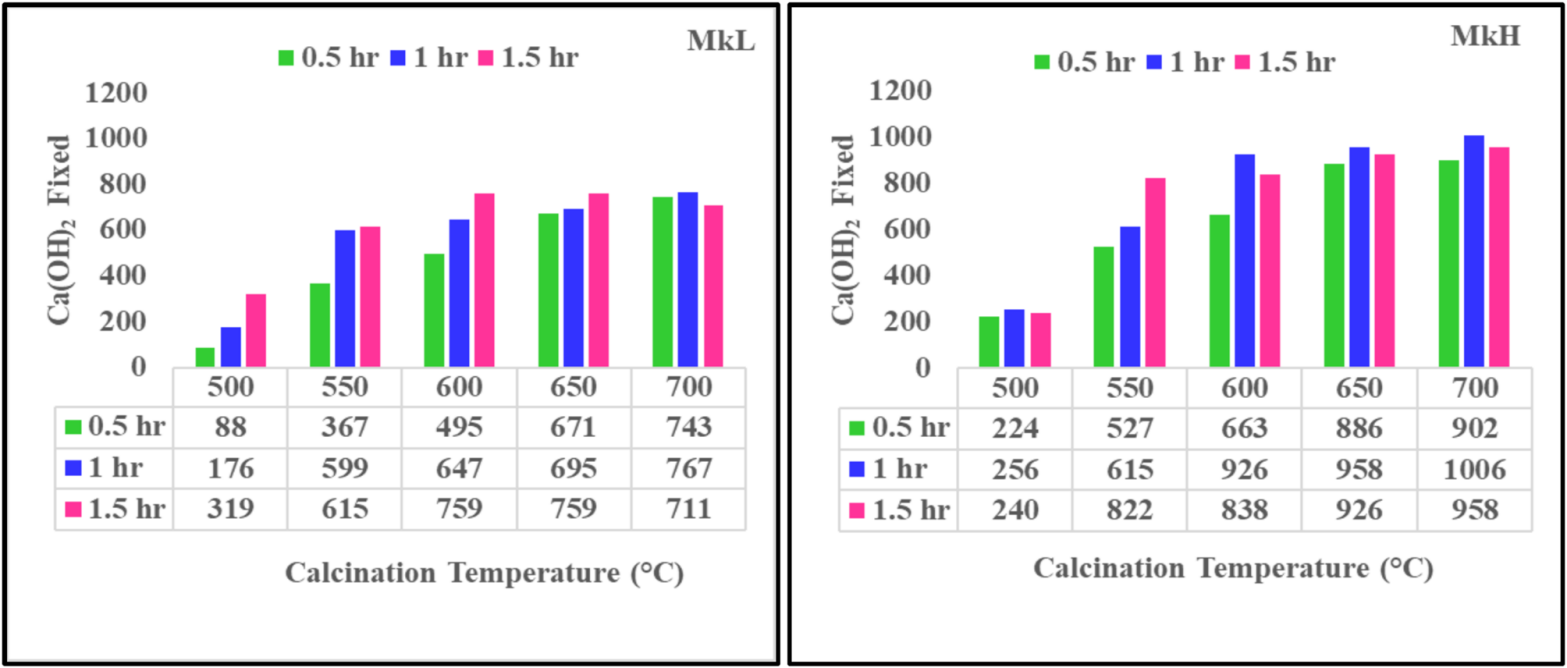
Pozzolanic activity of metakaolin samples.

The Chapelle test results for two metakaolins (MkL and MkH) prepared from calcination of corresponding kaolin samples (kL and kH) over a wide range of temperatures/periods (500, 550, 600, 650 and 700 °C for 0.5, 1 and 1.5 h) are shown in Fig. 3. The NF P 18–513^47^ specifies that the pozzolanic activity of metakaolin (Mk) must be at least 660 mg Ca(OH)_2_/g Mk to be used as a cementitious material. Figure 3 demonstrates that the amount of CaO consumed per 1 g Mk ranged from 88 to 767 for MkL and from 224 to 1006 for MkH. It is observed that heating kaolin at 500 °C for 0.5 h results in the lowest pozzolanicity. In contrast, the highest pozzolanic values for two metakaolins were recorded when the kaolin was heated at 700 °C for 1 h, which is consistent with the findings of the researchers^16,70–74^. Therefore, it can be concluded that calcining kaolin under this heating condition (700 °C/1h) causes its reorganization of structure through the loss of structural water^53^. Calcination of kaolin involves transforming inactive AlO_6_ octahedra into active tetra- or penta-coordinated units^53^. The different mineralogical origins of MkH and MkL may influence the distribution of Al coordination and the portion of these coordination states, as the hydroxylation process is sensitive to structural order, kaolinite crystallinity, and geological origin along with associated minerals or substitutions^75^. Izadifar et al.^76^ demonstrated that stacking layers greatly affect the dehydroxylation temperature, the number of dehydroxylation steps, and the volume changes of the minerals after dehydroxylation. They found that the dehydroxylation temperature of disordered kaolinite is less than that of ordered kaolinite. Mbey et al.^77^ suggested that the kaolinite’s crystallochemical parameters, including stacking faults and imperfections from chemical substitution influenced by geological origin, could impact its transformation into metakaolin and the subsequent dehydroxylation process. Johnston et al.^78^ studied the effect of the degree of crystallinity on the dehydroxylation of kaolinite and, consequently, on aluminium dissolution. They noted that a higher degree of disorder in the original kaolinite structure leads to a lower temperature needed for complete dehydroxylation. Also, based on the obtained data, they reported that complete dehydroxylation was required to render the aluminium soluble. They explained that during dehydroxylation, aluminium migrates to vacant sites previously occupied by hydroxyls, decreasing aluminium’s coordination number from VI (inactive unit) to V and IV (active units). Ding et al.^79^ reported that the metakaolin’s reactivity is highest when the proportion of Al(VI) is minimal, and the proportions of Al (IV and V) are maximal. These structure reorganizations and phase transformations processes reflect an increase in disorder (amorphous nature), which enhances pozzolanicity, promoting the geopolymerization process^50^. From another perspective, they have a significant impact on the compressive strength, as tetrahedral Al (IV) creates negative charges that are balanced by alkali cations (Na⁺), forming sodium-alumino-silicate-hydrate (N–A–S–H) as a strength-giving phase^80^. From these data (pozzolanic activity of MkL and MkH are 767 and 1006 Ca(OH)_2_/g Mk, respectively), it can be expected that the properties of geopolymers produced by MkH will be better than MkL. Finally, it can be concluded that although MkL and MkH have a comparable oxide composition, they can still vary in their reactive Al speciation. This is because XRF measures total Al but does not indicate how much of it converts to framework Al(IV) upon activation. Therefore, the efficiency of metakaolin in the preparation of geopolymer is not solely based on bulk oxide composition.

On the other hand, increasing the heating temperature and/or duration above optimal conditions decreases the tendency for disordering within the metakaolin structure^81^. In general, there is a strong relationship between the degree of amorphousness and reactivity (pozzolanicity); high amorphous content indicates high pozzolanic activity^82^.

#### Mineralogical and morphological analysis

XRF and XRD analyses for kaolin samples (kL and kH) and calcined ones (MkL and MkH) at the optimal conditions (700 °C/1h) were studied to examine the impact of the calcination process on the oxide and phase compositions. Table 2 shows that the MkH has a higher Al_2_O_3_ wt.% than MkL; they are 36.46 and 34.76%, respectively, compared to SiO_2_ wt.%, which are 51.46 and 56.18%, respectively. Also, L.O.I values for kL and kH (7.51 and 10.97%, respectively) confirm the value of Mmax measured by TGA (7.53 and 11.03%).

**Table 2:** Chemical composition of kaolin and metakaolin.

Oxide	SiO_2_	TiO_2_	Al_2_O_3_	Fe_2_O_3_	MnO	MgO	CaO	Na_2_O	K_2_O	P_2_O_5_	Cl	SO_3_	LOI
kL	62.27	1.87	26.91	0.98	< 0.01	0.07	0.09	< 0.01	< 0.01	< 0.01	< 0.01	< 0.01	7.51
MkL	56.18	2.63	34.76	1.46	0.02	0.21	0.97	< 0.01	< 0.01	< 0.01	< 0.01	0.11	3.37
kH	49.72	4.17	33.74	0.89	< 0.01	0.07	0.12	< 0.01	< 0.01	0.03	< 0.01	< 0.01	10.97
MkH	51.46	5.22	36.46	1.24	0.03	0.20	1.24	< 0.01	< 0.01	< 0.01	< 0.01	0.31	3.55

**Fig. 4:**
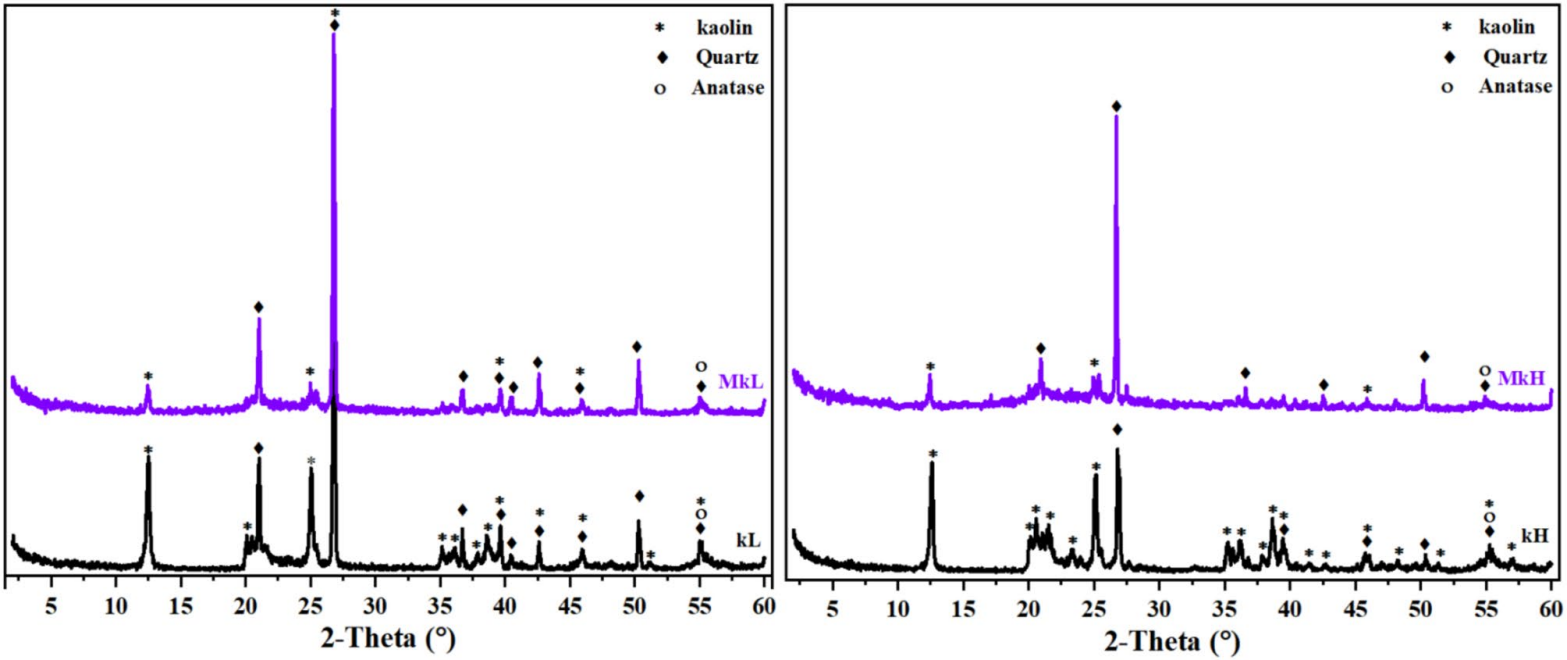
XRD patterns of kaolin samples before and after calcination at 700 °C for 1 h.

In the XRD analysis (Fig. 4), kaolinite was identified alongside quartz and minor amounts of anatase in the kaolin samples. The results show that the amount of kaolinite phase in kH is higher than in kL, which is compatible with TGA/DTG analysis. Furthermore, the widespread peaks of kaolinite in type kH indicate its lower degree of crystallinity referring to the possibility of producing metakaolin with higher pozzolanic activity than type kL and thus producing geopolymers with better properties. The XRD patterns for the calcined kaolins, shown in Fig. 4, indicate that the characteristic reflections of kaolinite from MkL and MkH were eliminated at the following 2θ: 20.6°, 35.2°, 36.7°, 37.9°, 38.6°, 42.6°, 51.2°, and 55.0° for MkL; and 20.2°, 20.6°, 21.5°, 23.4°, 35.3°, 36.1°, 38.7°, 39.5°, 45.8°, 51.4°, and 55.3° for MkH. This suggests a transformation from kaolin to metakaolin, as well as a significant decrease in crystallinity, occurring at a temperature of 700 °C^66,83,84^. The reduction in reflection intensity at 2θ = 12.5° for MkL and at 2θ = 12.6, 25.1, 48.1° for MkH indicates a breaking of bonds between kaolinite layers^84^. The diffractogram of the MkL and MkH clearly shows the unchanged presence of quartz reflections, similar to those in anatase. These results show that the XRD analysis aligns with the degree of dehydroxylation and Chapelle results, indicating kaolin activation at 700 °C.

**Fig. 5:**
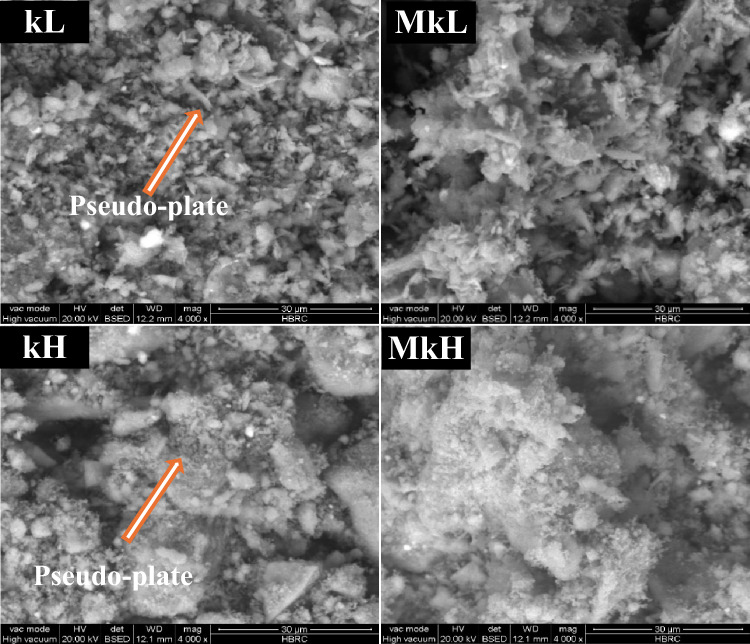
SEM-images of kaolin samples before and after calcination at 700 °C for 1 h.

The data obtained from XRD analysis was also confirmed by SEM (Fig. 5). The presence of kaolinite in the kL and kH kaolin samples is clearly visible in SEM images. Conversely, this phase disappeared after thermal treatment. Heat treatment at 700 °C for the kaolin samples kL and kH did not alter the overall platy morphology of the phases. However, the particle edges became more rounded and the agglomeration of platelets into irregular stacks became more noticeable. Based on the above discussion, it is concluded that calcination at 700 °C for 1 h transformed the crystalline kaolin into a more reactive aluminosilicate precursor better suited for geopolymerization.^72,85^.

From all the previous results, it can be concluded that the kaolin calcined at 700 °C for 1 h had the highest pozzolanic value because it contained the greatest amount of amorphous material. This finding aligns with previous studies^66,84^. Souri et al.^84^ calcined kaolin at different temperatures from 600 to 850 °C for 3 h. They concluded that calcination at 700 °C is the optimum firing temperature (the highest amorphousness), beyond this temperature, metakaolin recrystallized. Another study used the measured heat evolved during the polycondensation process to measure the optimum firing conditions. Cioffi et al.^86^ found that the heat evolved is low in the case of kaolin fired at 550 °C for 2 h, while it was very high in the case of firing at 650 °C for the same period. From these results, they predicted that firing kaolin at 650 °C for 2 h is the optimum condition among firing at 500, 550, 650 and 700 °C for 2, 4 and 6 h. There is a wide variation in the optimum calcination temperature of kaolin due to its dependence on the crystallinity of the original kaolinite^43^.

### Fresh and hardened properties of

To study the effect of the mineralogical composition of metakaolin samples on the fresh and hardened properties of metakaolin-based geopolymers, the workability, setting time and compressive strength of the prepared specimens, derived from the activation of MkL and MkH by NaOH and Na_2_SiO_3_ were measured. Accordingly, different geopolymers with the same molar ratios (SiO_2_/Al_2_O_3_, Al_2_O_3_/Na_2_O and H_2_O/Na_2_O) were prepared from two metakaolin samples, as tabulated in Table 3. In the mixes-design (Table 3), the specimens prepared from MkL and MkH were coded as GL and GH, respectively. MkL has a higher percentage of initial silica in its chemical composition (initial SiO_2_/Al_2_O_3_= 2.7), while MkH has a lower percentage (initial SiO_2_/Al_2_O_3_= 2.4). The molar ratio of SiO_2_/Al_2_O_3_ required for each group was adjusted by adding Na_2_SiO_3_ solution to the metakaolin. So, MkL requires adding a smaller amount of Na_2_SiO_3_ solution than MkH to achieve the desired SiO_2_/Al_2_O_3_ molar ratios in the geopolymer paste. In the 1st group in Table 3, Na_2_SiO_3_ solution weights of 72.9 and 150.7 g were added to the mixes# GL1 and GH1, respectively, to achieve a molar SiO_2_/Al_2_O_3_ ratio of 3.1.

**Table 3:** Mix-design used in the preparation of geopolymer.

Mix. No	MK mass (gm)	NaOH solution mass (gm)	Na_2_SiO_3_ solution mass (gm)	#M NaOH (molar)	Liquid/Solids	Na_2_SiO_3_/ NaOH	SiO_2_/ Al_2_O_3_	Al_2_O_3_/Na_2_O	H_2_O/ Na_2_O	Water/solids
GL1	300	201.4	72.9	9.01	0.91	0.36	3.10	1.20	13.0	0.533
GH1	300	159.5	150.7	10.05	1.03	0.95	3.10	1.20	13.0	0.522
GL2	300	173.0	113.7	9.54	0.96	0.66	3.30	1.20	13.0	0.516
GH2	300	129.5	193.6	11.03	1.08	1.50	3.30	1.20	13.0	0.506
GL3	300	144.5	154.4	10.27	1.00	1.07	3.50	1.20	13.0	0.500
GH3	300	99.7	236.3	12.59	1.12	2.37	3.50	1.20	13.0	0.491
GL4	300	166.5	195.3	10.46	1.21	1.17	3.70	1.00	13.0	0.567
GH4	300	122.5	279.2	12.43	1.34	2.28	3.70	1.00	13.0	0.557

#### Fresh properties

Generally, the fresh behavior of metakaolin-based geopolymers depends on the evaluation of workability and setting time, and it directly influences the feasibility of casting, compaction, and finishing. In alkali-activated systems, these properties are influenced by the alkaline activator type/concentration, which affects the kinetics of the geopolymerization process.

Regarding workability, increasing the water/solids ratio usually enhances the initial workability of alkali-activated binders because it reduces solids packing and interparticle friction^87^. Table 4 shows an evident variation in observed workability during the casting process, ranging from high for GL1 and GH1 to medium for GL2 and GH2, then low for GL3, GH3, GL4 and GH4. This trend aligns with the systematic reduction in water/solids in the 1st, 2nd and 3rd group^87^. Also, it is noticed that although the 4th group has the highest water/solids ratio, it has low workability. This proves that water content alone does not entirely determine workability; the activator’s type/concentration has a great impact, as the activator can both raise solution viscosity and speed up structural formation^88^. Across the mix-design, NaOH molarity and Na_2_SiO_3_/NaOH ratio increased from 9.01 M and 0.36 in GL1 up to 12.43 M and 2.28 in GH4, respectively. Fresh metakaolin geopolymer pastes are greatly affected by the viscosity of the Na_2_SiO_3_ solution, as well as dissolution/condensation kinetics that are controlled with activator concentration. Therefore, increasing Na_2_SiO_3_ dosage and NaOH molarity generally results in higher viscosity, which aligns with the observed low workability classifications at high Na_2_SiO_3_/NaOH ratios and high NaOH molarity^89^.

**Table 4:** Initial and final setting time and observed workability of geopolymer.

Mix. No	Initial setting time (mins)	Final setting time (mins)	Observed workability
GL1	88	146	High
GH1	966	1305	High
GL2	48	108	Medium
GH2	622	915	Medium
GL3	20	31	Low
GH3	238	357	Low
GL4	10	15	Low
GH4	143	259	Low

On the other hand, the initial/final-setting time (I/F-ST) results cover a wide range, from very fast setting (GL4: 10/15 min and GL3: 20/31 min) to very slow setting (GH1: 966/1305 min and GH2: 622/915 min). This variation is not only controlled by SiO_2_/Al_2_O_3_ but also by the rate of dissolution, which is affected by alkali content. It can be related the long setting times of GH1 and GH2 to their much higher Na_2_SiO_3_/NaOH ratio compared with the GL1 and GL2 mixes at a constant SiO_2_/Al_2_O_3_; at SiO_2_/Al_2_O_3_ of 3.1 and 3.3, the Na_2_SiO_3_/NaOH ratio of GH1 and GH2 are 0.95 and 1.5 and for GL1 and GL2 are 0.36 and 0.66, respectively. A pore solution rich in SiO_2_ can maintain a viscous, polymerized silicate environment that hinders the development of a load-bearing gel network, thus delaying I/F-ST even when the paste is initially workable^35^. Conversely, across the groups, the reduction in the I/F-ST indicates rapid geopolymerization, which is attributed to alkali availability that causes fast dissolution and condensation kinetics^90^.

#### Compressive strength

**Fig. 6:**
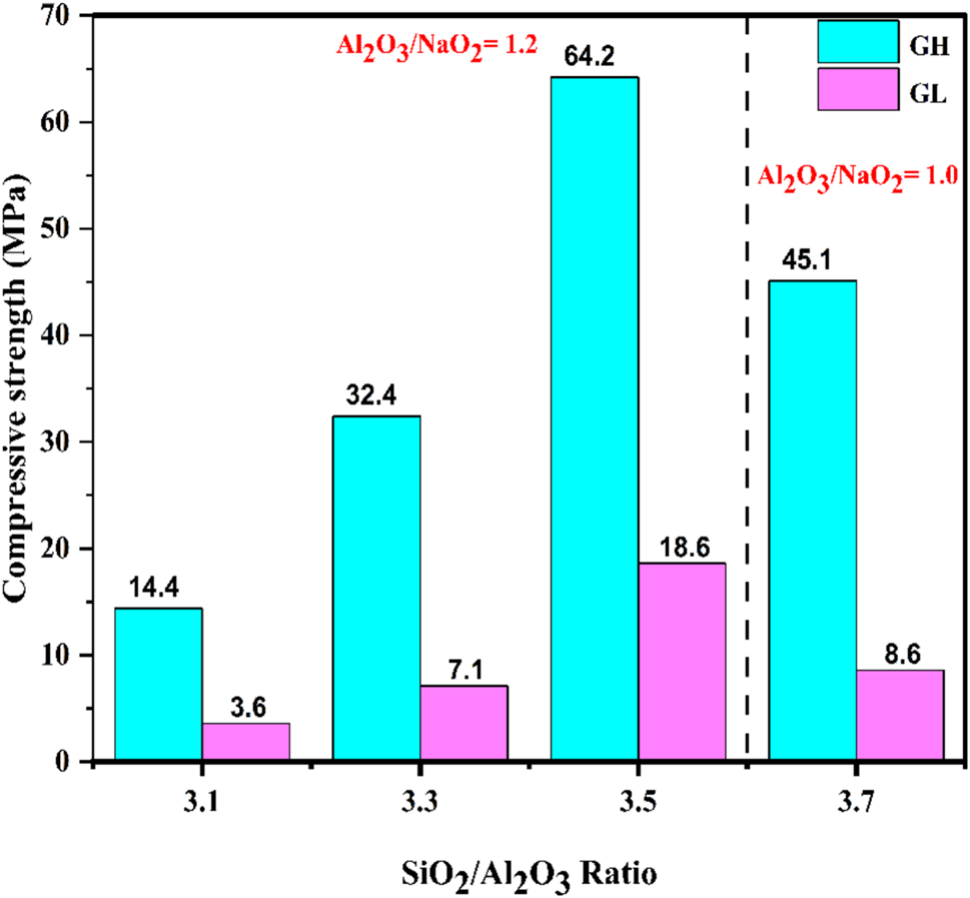
Compressive strength of MkL and MkH-based geopolymers at 28-days.

The compressive strength of building materials shows their availability to be used in structural applications; therefore, the compressive strength of the prepared metakaolin-based geopolymer was measured, as shown in Fig. 6. The compressive strength achieved for the mix# GL1 was 3.6 MPa, while it was 14.4 MPa for the mix# GH1. This pattern was also repeated in the next three groups from Table 3, where the compressive strength was greater in the mixes containing a larger amount of Na_2_SiO_3_ solution, even though they had the same three molar ratios SiO_2_/Al_2_O_3_, Al_2_O_3_/Na_2_O, and H_2_O/Na_2_O. Therefore, the amount of dissolved silica available from Na_2_SiO_3_ solution played a key role in the geopolymerization process and in developing compressive strength.

As in Fig. 6, a clear trend indicates that the compressive strength initially increases with rising SiO_2_/Al_2_O_3_ ratio. It continues to grow until reaching a peak at a ratio of 3.5, then decreases as the SiO_2_/Al_2_O_3_ ratio increases further. This pattern was observed in both geopolymers produced from two different types of metakaolin (MkH and MkL). The maximum compressive strength of 64.2 MPa was achieved for the GH, while 18.6 MPa was obtained for the GL. Geopolymer specimens with low SiO_2_/Al_2_O_3_ molar ratios tend to have low strength. This may be due to the formation of poly(sialate) polymers during geopolymerization, creating a weak and brittle structure with limited hydraulic properties. A low SiO_2_/Al_2_O_3_ ratio produces a less cross-linked, two-dimensional network, which is less durable and weaker^35^. In contrast, increasing the SiO_2_/Al_2_O_3_ ratio promotes the dominance of poly(sialate-siloxo) and poly(sialate-disiloxo) frameworks within the geopolymer matrix, which are stronger than poly(sialate) alone, thereby enhancing the overall structural integrity of the geopolymer^35,86^. A further increase in SiO_2_/Al_2_O_3_ within the geopolymeric matrix saturates the bulk solution with a large amount of SiO_2_, hindering the dissolution of metakaolin^35,40^. This indicates a significant effect of the SiO_2_/Al_2_O_3_ ratio on the compressive strength of Metakaolin-based materials geopolymers. Similar findings have been reported in previous studies by Silva et al.^35^, Kamalloo et al.^40^ and Kuenzel et al.^91^.

It is also noticed that the MkH-based geopolymer has a compressive strength higher than that prepared from MkL. It is higher by 300, 413.4, 245.2 and 424.4% at SiO_2_/Al_2_O_3_ of 3.1, 3.3, 3.5 and 3.7, respectively. The main reason behind this behavior may be due to the high pozzolanic activity of MkH compared to MkL. Furthermore, the high amount of active silica added to the matrix by incorporating Na_2_SiO_3_ in the case of using MkH may be another reason for its high compressive strength, as shown above. Table 3 shows that the SiO_2_/Al_2_O_3_ ratio increased from 3.1 to 3.7 in both metakaolin sources (MkL and MkH), as the SiO_2_ contribution from Na_2_SiO_3_ increased, accompanied by an increase in the compressive strength values (optimum ratio was SiO_2_/Al_2_O_3_ of 3.5). This indicates that soluble SiO_2_ supplied by Na_2_SiO_3_ strongly controls strength development. The XRF analysis demonstrated that MkL contains a total SiO_2_ wt.% higher than MkH; however, MkL has a lower strength than MkH across all ratios. This refers to that the total SiO_2_ in the chemical composition of the precursor is usually not considered a predictor of strength. Since a portion of SiO_2_ in raw kaolin may exist as residual quartz (less reactive forms), and this has been confirmed by the XRD analysis (Fig. 4), which shows that the quartz peak intensity in MkL is higher than in MkH. These findings support the conclusion that strength is more closely linked to the amount of soluble silicate (from Na_2_SiO_3_) available than to the total SiO_2_ content of the precursor measured by XRF. Also, it is detected that although both metakaolin sources reach maximum compressive strength at SiO_2_/Al_2_O_3_ of 3.5, their sensitivity to further silica increase varies significantly. MkL shows a more pronounced strength loss at SiO_2_/Al_2_O_3_ of 3.7, likely due to buffer capacity effects. Generally, the buffer capacity of an aluminosilicate precursor in geopolymer refers to its ability to incorporate ions/species supplied by the alkaline solution (Na^+^, OH^-^ and SiO_2_) into the formation of geopolymeric products (N–A–S–H), rather than leaving an excess amount in the pore solution, which can affect the compressive strength^92^. The buffer capacity influences the reactivity of the material, the solubility of silica and alumina, and the overall kinetics of the geopolymerization reaction^92^. Based on pozzolanic activity differences, the higher buffer capacity of MkH can be discussed. MkL and MkH show pozzolanic activity of 767 and 1006 mg Ca(OH)₂/g, respectively. The higher pozzolanicity of MkH indicates that it can generate more reactive Al-Si species, making it has a great ability to adapt to higher alkalinity/soluble-silicate with less loss of compressive strength^93^. On the other hand, the low pozzolanicity of MkL refers to a lack of reactive Al species, leading to silica overload, where excess silicate is not effectively incorporated into the gel network and instead results in unreacted phases, increased porosity and weaker binding^35,40^. Accordingly, it can be reported that the compressive strength achieved varies depending on the chemical composition of the raw kaolin and the extent of metakaolin’s amorphousness resulting from calcination. This conclusion is consistent with previous studies that report different maximum strengths for various kaolin types and SiO_2_/Al_2_O_3_ ratios. Ozer et al.^39^ used Turkish kaolin and calcined it at 700 °C for 1 h, achieving an optimal strength of 23 MPa at a SiO_2_/Al_2_O_3_ ratio of 4.4. While Mo et al.^38^ used Chinese kaolin and calcined it at 800 °C for 3 h, reaching an optimal strength of 98.0 MPa at a SiO_2_/Al_2_O_3_ ratio of 3.3. In addition, Ayeni et al.^12^ used Nigerian kaolin and calcined it at 700 °C for 2 h, obtaining an optimal strength of 10.0 MPa at a SiO_2_/Al_2_O_3_ ratio of 3.1. It must be noticed that the 4th group should not be interpreted as a direct extension of the single-parameter SiO_2_/Al_2_O_3_ series, as Al_2_O_3_/Na_2_O changes from 1.2 in the 1st, 2nd and 3rd groups to 1.0. Since the alkali dosage significantly affects the geopolymerization kinetics, and consequently the strength progression of the prepared specimens.

### Statistical study

A statistical study using ANOVA analysis was conducted to investigate how factors such as kaolin type, calcination temperature/period affect the pozzolanic activity of corresponding metakaolin powder, as well as the properties of geopolymer, including metakaolin pozzolanicity and molar ratios in the mix design. The ANOVA results, including F-values and *p* value, were considered statistically significant for the response factor at a 95% confidence level. The significance of each factor’s relative importance is evaluated using F-values; an F > Fcrit indicates a significant effect of that factor. Moreover, when the *p* value falls below the significance threshold of α = 0.05, it confirms the factor’s statistical significance. The ANOVA results in Tables 5, 6 and 7 reveal that for all investigated factors, F > Fcrit and the *p* value < 0.05, demonstrating that all the parameters analyzed are statistically significant^50,94–96^. From these data, it can be detected that the metakaolin source significantly affects both precursor reactivity and geopolymer properties. As shown in Table 5, calcination temperature/period has a highly significant effect on pozzolanic activity for metakaolin types. Most importantly, under the same calcination conditions (700 °C for 1 h), the metakaolin types (MkL and MkH) also show a highly significant difference in pozzolanic activity. Consistently, Tables 6 and 7 confirm that mix design significantly affects setting time and compressive strength within each geopolymeric system (GL and GH), and that the metakaolin type produces a statistically significant difference in these properties even at a constant SiO_2_/Al_2_O_3_ ratio. Therefore, the main reasons for the differences are variations in metakaolin reactivity rather than in bulk oxide composition.

**Table 5:** ANOVA result for pozzolanic activity.

Factor	Condition	SS		*df*		MS		F	*P* value	F-crit	Significance criteria
		Between groups	Within groups	Between groups	Within groups	Between groups	Within groups				
Effect of different calcination temperatures at a constant duration for MkH	500, 550, 600, 650 and 700 °C for 1 h	1,205,046	5285.4	4	10	301,261.4	528.54	569.98	7.6E-12	3.48	Significant
Effect of different calcination temperatures at a constant duration for MkL	500, 550, 600, 650 and 700 °C for 1 h	644,767	3953	4	10	161,191.8	395.26	407.81	5.0127E-11	3.48	Significant
Effect of different calcination periods at a constant temperature for MkH	700 °C for 0.5, 1 and 1.5 h	17,054	683	2	6	8527.21	113.78	74.942	0.000031	5.14	Significant
Effect of different calcination periods at a constant temperature for MkL	700 °C for 0.5, 1 and 1.5 h	5558	1201	2	6	2779.11	200.11	13.888	0.0091	5.14	Significant
Effect of different Metakaolin types at constant calcination temperature/period	MkL and MkH at 700 °C for 1 h	1,666,210	561	1	4	1,666,210	140.17	11,887.35	1.3E-08	7.71	Significant

**Table 6:** ANOVA result for final setting time.

Factor	Condition	SS		*df*		MS		F	*P* value	F-crit	Significance criteria
		Between Groups	Within Groups	Between Groups	Within Groups	Between Groups	Within Groups				
Effect of different mix designs of GL Geopolymer	GL1, GL2, GL3 and GL4	48,536	71	3	8	16,178.8	8.916	1814.445	4.92E-09	4.07	Significant
Effect of different mix designs of GH Geopolymer	GH1, GH2, GH3 and GH4	3,377,986	473	3	8	1,125,995	59.083	19,057.75	2.51E-09	4.07	Significant
Effect of different metakaolin types (MkL and MkH) at constant SiO_2_/Al_2_O_3_ ratio	GL3 and GH3	294,811	85	1	4	294,810.7	21.166	13,928.06	1.17E-06	7.71	Significant

**Table 7:** ANOVA result for mechanical compressive strength at 28 days.

Factor	Condition	SS		*df*		MS		F	*P* value	F-crit	Significance criteria
		Between groups	Within groups	Between groups	Within groups	Between groups	Within groups				
Effect of different mix designs of GL Geopolymer	GL1, GL2, GL3 and GL4	587	8	3	8	195.6098	0.945	206.9945	4.92E-09	4.07	Significant
Effect of different mix designs of GH Geopolymer	GH1, GH2, GH3 and GH4	7618	84	3	8	2539.408	10.533	241.083	2.51E-09	4.07	Significant
Effect of different metakaolin types (MkL and MkH) at constant SiO_2_/Al_2_O_3_ ratio	GL3 and GH3	9289	59	1	4	9288.891	14.856	625.2338	1.17E-06	7.71	Significant

### Phase identification

#### XRD analysis

**Fig. 7:**
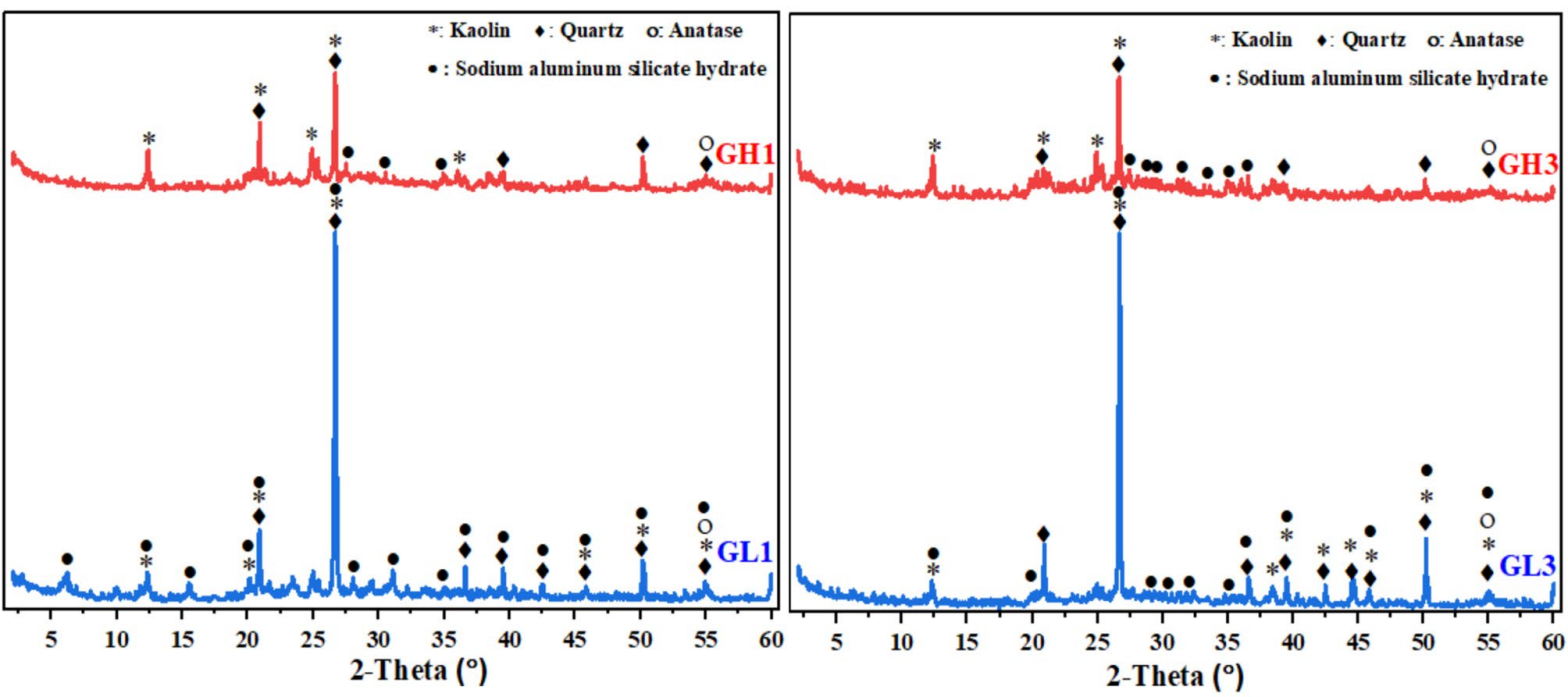
XRD patterns of different geopolymeric specimens at 28-days.

The phase components of the two geopolymer types (GL and GH) at SiO_2_/Al_2_O_3_ ratios of 3.1 and 3.5 after 28 days of curing are shown in Fig. 7. The XRD pattern for all specimens shows sharp peaks indicating the presence of trace kaolin, quartz, and anatase in the original clay. These materials are inactive and not influenced by the alkali-activation process^97,98^. At SiO_2_/Al_2_O_3_ = 3.1 (mixes# GL1 and GH1), there was a distinct peak distribution in the range of 2θ = 20–35°. This indicated that the sodium-alumino-silicate-hydrate gel (N–A–S–H), which is the main strength-giving-phase in the geopolymerization process^99,100^, existing in an amorphous form. As the SiO_2_/Al_2_O_3_ ratio increased to 3.5, the peaks related to N–A–S–H became more prominent, indicating proper geopolymerization and an increase in the amount of formed N–A–S–H gel. Since both molar ratios (Al_2_O_3_/Na_2_O and H_2_O/Na_2_O) used in the preparation of specimens remained constant, it can be concluded that the variations in the SiO_2_/Al_2_O_3_ ratio are one of the main factors that influence the reaction levels. This matches the compressive strength results shown in Fig. 6, which gradually increased with the SiO_2_/Al_2_O_3_ ratio until reaching a peak, then declined as the ratio continued to increase.

Figure 7 also shows variations in the intensity of the peaks for geopolymers within the same group (mixes# GL1/GH1 and GL3/GH3), despite the constant of all molar ratios. This indicates that the variation in mineralogical composition and subsequent pozzolanic activity of the two metakaolin samples used in geopolymer production contributed to the differences in phase composition within the geopolymeric matrix. Besides the high pozzolanic activity of MkH, which indicates that it contains a greater quantity of reactive silicate species compared to MkL, the larger quantity of Na_2_SiO_3_ required for MkH to achieve the target molar ratios provides a large quantity of dissolved silicate species, and this may be the reason behind these differences. In the 1st group, 72.9 and 150.7 g of Na_2_SiO_3_ solution were added to types GL1 and GH1, respectively, to achieve a SiO_2_/Al_2_O_3_ ratio of 3.10. In the third group, 154.4 and 236.3 g of Na_2_SiO_3_ solution were added to types GL3 and GH3, respectively, to achieve a SiO_2_/Al_2_O_3_ ratio of 3.50. Generally, the silicate supplied by Na_2_SiO_3_ is present in the geopolymeric matrix as dissolved species, which can participate directly in the network development. On the other hand, the silicate species in metakaolin must first be released by dissolution before contributing to the gel formation. Finally, it can be concluded that the difference may be correlated with the silicate availability and precursor dissolution kinetics.

#### TGA/DTG analysis

**Fig. 8:**
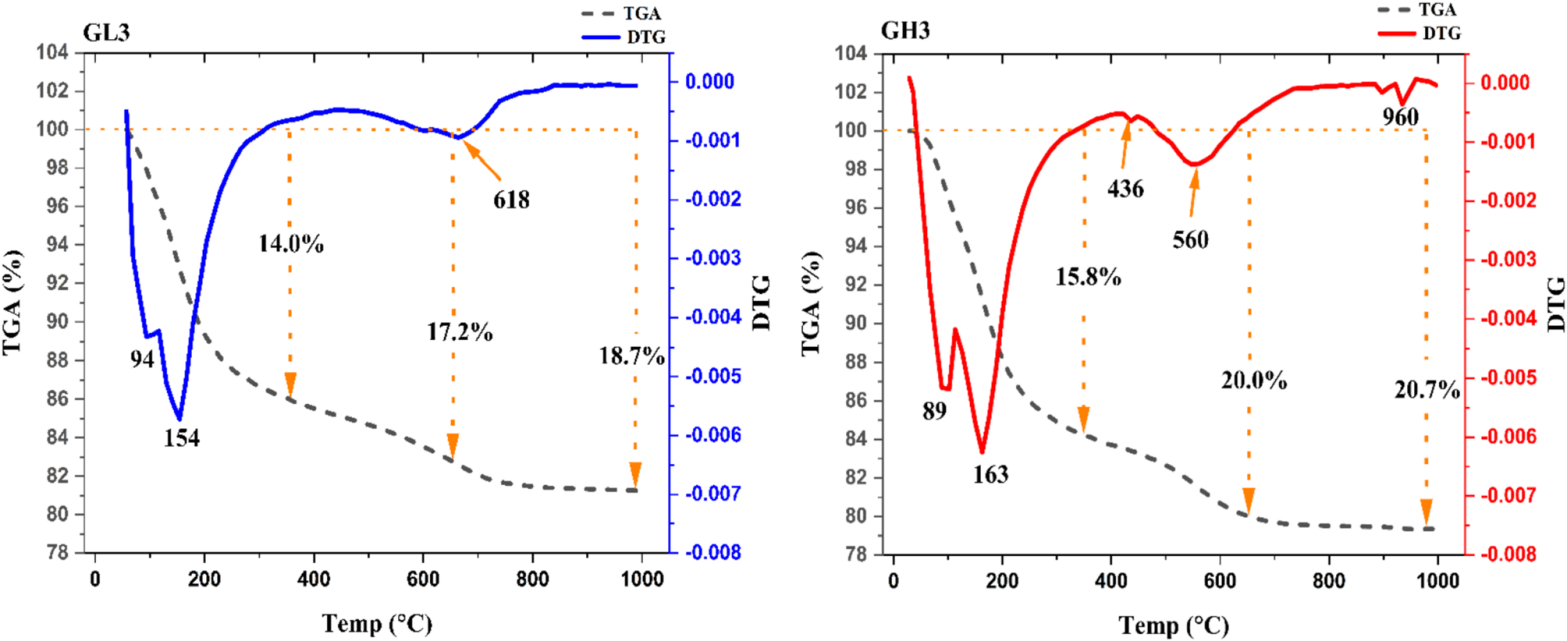
TGA/DTG analysis GL3 and GH3 specimens at 28-days.

Qualitative and quantitative analyses for the phase composition of two geopolymeric specimens (mixes# GL3 and GH3) at 28-days were examined using thermogravimetric analysis (TGA) with differentiation (DTG), as represented in Fig. 8. The thermograms of the two specimens show two endothermic peaks ranging from ambient temperature to 200 °C, accompanied by a sharp decrease in mass, which is caused by the release of both free water and some tightly bound water from the geopolymer structure^101,102^. The mass loss up to 200 °C is approximately 10.8 and 11.8wt.% for the two samples, accounting for about 58 and 57%, respectively, of the total mass loss for mixes# GL3 and GH3. Notably, the main endothermic peak below 200 °C for the mix# GH3 appears at a higher temperature (163 °C) than for mix# GL3 (154 °C). This demonstrates that the presence of silica as an active soluble liquid (Na_2_SiO_3_) was more effective than its presence in the chemical composition of metakaolin. Increasing the amount of soluble silica (Na_2_SiO_3_) leads to the formation of a stronger bond within the geopolymer gel. As a result, a denser matrix was produced. This aligns with the compressive strength of the mix# GH3, which reached 64.2 MPa, compared to mix# GL3, which achieved a compressive strength of 18.6 (Fig. 6). The geopolymers continue to lose mass up to 350 °C, with a total loss of approximately 14.0% and 15.8% for mixes# GL3 and GH3, respectively. This may be due to the dehydroxylation process in the geopolymers. The dehydroxylation process persists between 350 and 650 °C, resulting in a gradual mass loss of approximately 3.2% to 4.2% in mixes# GL3 and GH3, respectively^103^. It is evident that the endothermic peak around 560 °C for the geopolymer mix# GH3 is more prominent than that of mix# GL3 at approximately 618 °C, indicating a higher amount of N–A–S–H in mix# GH3, which was prepared from highly pozzolanic metakaolin (MkH). The endothermic peak detected in the mix# GH3 at 436 °C corresponds to the dehydroxylation of the aluminosilicate gel and the remaining kaolinite. Finally, the mass loss% observed beyond 650 °C reaches 18.7 and 20.7% for mixes# GL3 and GH3, respectively, may result from the decomposition of carbonated phases due to exposure of the specimens to atmospheric CO_2_^104–106^.

### Microstructure analysis

#### SEM analysis

**Fig. 9:**
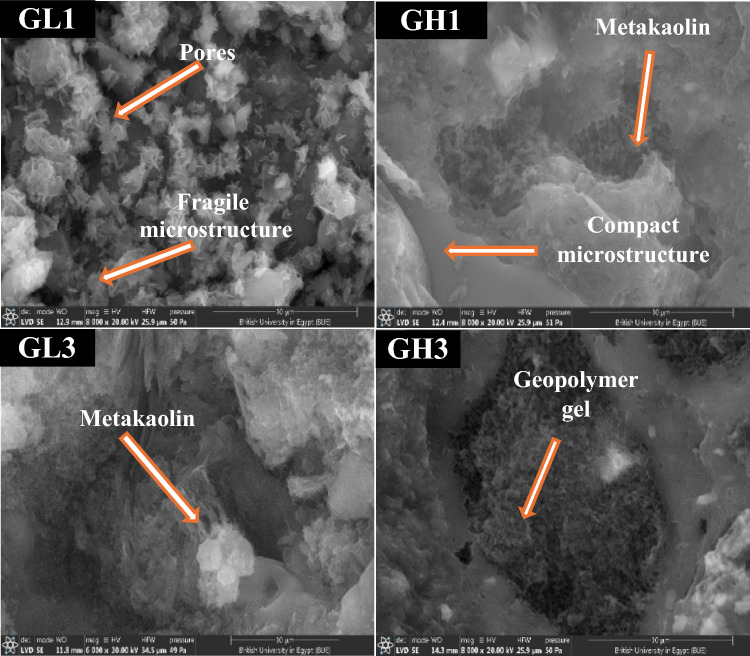
SEM images of some selected specimens at 28-days.

After 28 days of hydration, SEM analysis of four geopolymers (mixes # GL1, GH1, GL3, and GH3), as represented in Fig. 9. It was conducted to compare the microstructural features of hardened paste specimens prepared with (i) different sources of metakaolin (MkL and MkH); and (ii) the same molar ratio of SiO_2_/Al_2_O_3_, using different amounts of soluble silica (Na_2_SiO_3_). In geopolymer specimens with a SiO_2_/Al_2_O_3_ ratio of 3.1, Mix# GL1, which was activated with the lowest amount of Na_2_SiO_3_ (72.9 g), shows a fragile microstructure with wide pores containing unreacted metakaolin and needle-like crystals of geopolymeric products. This indicates that the amounts of dissolved aluminate and silicate species from metakaolin and added from Na_2_SiO_3_ were insufficient to form the geopolymeric products, suggesting an incomplete alkali-activation process. Consequently, it has the lowest compressive strength (3.6 MPa). However, increasing the Na_2_SiO_3_ amount from 72.9 to 150.7 g for the same SiO_2_/Al_2_O_3_ ratio of 3.1, as in mix# GH1, creates a compact structure, enhancing the compressive strength (14.4 MPa).

Figure 9 illustrates the efficacy of augmenting the molar ratio of SiO_2_/Al_2_O_3_ from 3.1 to 3.5 accompanied by increasing Na_2_SiO_3_ mass. This escalation is congruent with an increase in compressive strength of geopolymers, as it promotes the polymerization process^29^. In sample GL3, which has a SiO_2_/Al_2_O_3_ ratio of 3.5, the amount of unreacted metakaolin was significantly decreased. Therefore, the overall microstructure of the gel matrix became dense and with few pores due to the bonding of metakaolin particles with formed zeolitic products, resulting in the development of the compressive strength values^107^. The compressive strength of GL3 increased by 345.8%, with respect to GL1. Comparing GL3 with GH3, maintaining a consistent SiO_2_/Al_2_O_3_ ratio requires increasing the Na_2_SiO_3_ mass from 154.4 to 236.3 g. This increment facilitates the transformation of metakaolin into a geopolymeric gel that fills the pores, forming a compact matrix.

#### Textural characteristics

The BET and BJH models were used to examine the impact of variation in mineralogical composition of metakaolin and the resulting change in Na_2_SiO_3_ mass to maintain a constant SiO_2_/Al_2_O_3_ ratio on the microstructure and textural properties of the produced metakaolin geopolymer specimens. After 28 days of hydration, a comprehensive textural analysis was conducted using the N_2_ adsorption/desorption technique on the mixes# GL3 and GH3. The main textural features, such as specific surface area (S.A, m^2^/g), maximum pore diameter (dpmax, nm), total pore volume (Vt, cm^3^ /g), monolayer capacity (Vm, cm^3^/g) and median pore diameter were determined to describe the nature of porosity^108,109^.

**Fig. 10:**
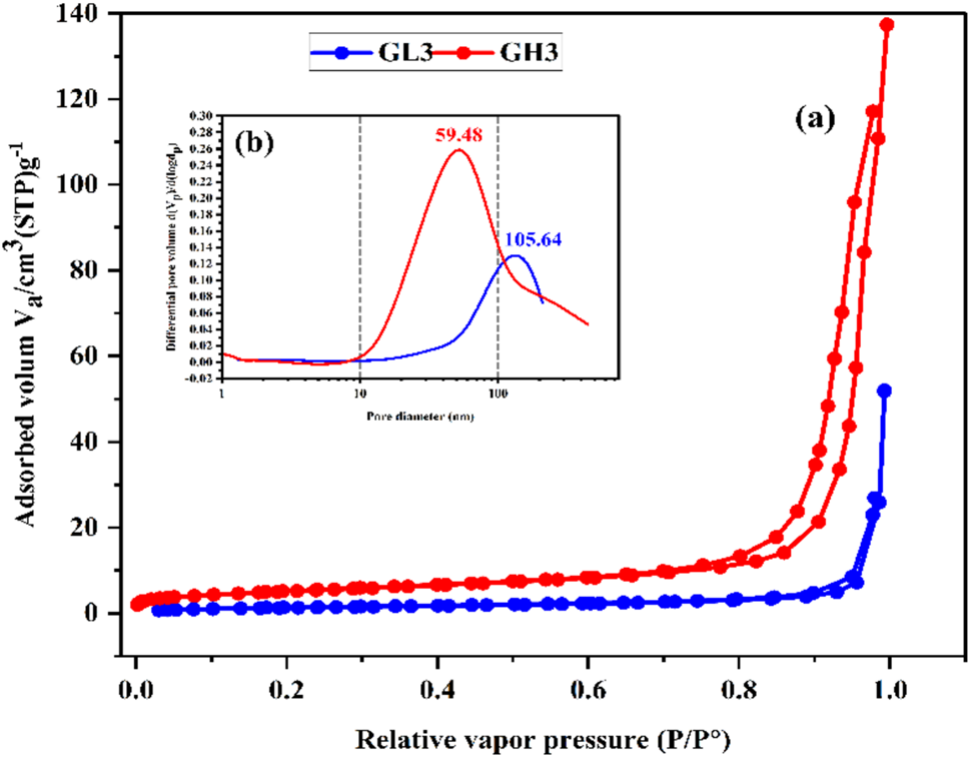
(**a**) N2-adsorption/desorption isotherms and (**b**) Pore size distribution for mixes# GL3 and GH3 at 28 days of hydration.

Referring to Fig. 10 and Table 8, mixes# GL3 and GH3 display a type IV isotherm with H3 hysteresis. Generally, the type IV isotherm is associated with mesostructured solids, where adsorption occurs at intermediate relative pressures, followed by capillary condensation. Simultaneously, H3 hysteresis is commonly related to the formation of slit‑shaped pores. In metakaolin-based geopolymers, this slit-like porosity aligns with the development of a gel network (N‑A‑S‑H) composed of fine geopolymeric products and inter-grain voids. As shown in Table 8, GH3 has a significantly higher S.A. of 18.46 m^2^/g compared to GL3’s 5.02 m^2^/g, along with Vm values of 4.24 and 1.15 cm^3^/g, respectively. In physisorption, an increase in S.A and Vm indicates a larger accessible internal surface area and the presence of more adsorption sites. This aligns with the development of the gel microtexture and/or finer internal interfaces within the geopolymeric products. The higher Vt of GH3 at 0.190 cm^3^/g compared to GL3’s 0.063 cm^3^/g further suggests that the GH3 matrix has a greater volume of pores accessible to N_2_ adsorption. Mohsen et al.^110^, Ramadan et al.^111^ and Amin et al.^112,113^ reported that an increase in the total pore volume refers to the elimination of macropores, strongly indicating the development of mesoporous structure augmented with strength-giving phases that strengthened the microstructure. Accordingly, the increase in the total pore volume doesn’t necessarily mean deterioration in the microstructure. Instead, it indicates that large macropores are being transformed into numerous smaller mesopores generated through the proper geopolymerization process. This can discuss the enhancement in the compressive strength for GH3 over GL3. Also, in physisorption, the pore-size distribution (PSD) is usually described by dpmax and D50. The dpmax refers to the most probable pore diameter at which the distribution reaches its maximum, not the largest pore diameter present. On the other hand, the D50 is the pore diameter at which the cumulative pore volume reaches 50% of the total pore volume, meaning that 50% of the cumulative pore volume occurs at pore diameters ≤ D50, and the remaining 50% occurs at diameters > D50. The PSD reveals that GH3 exhibits a more refined pore structure relative to GL3. The peak of PSD (dpmax) shifts from 105.64 nm in GL3 to 59.48 nm in GH3, implying that the dominant accessible pore size moves toward smaller diameters. In addition, the D50 decreases from 88.12 nm for GL3 to 41.25 nm for GH3. According to the IUPAC system classifications, they categorized the pores into three categories: micropores with dpmax < 2 nm, mesopores with dpmax 2–50 nm, and macropores with dpmax > 50 nm^114^. The PSD indicates a larger fraction of GH3’s pore volume in the mesopore range, while GL3 exhibits a greater contribution from pores > 50 nm. Overall, the peak shift (dpmax) and D50 indicate that GH3 possesses a more refined pore compared to GL3. From the previous data, it can be concluded that GH3 has higher S.A, Vm and Vt, as well as a smaller pore size (D50 and dpmax), reflecting the presence of a large fraction of fine pores within the gel products rather than macro-defect porosity.

**Table 8:** The main textural parameters of GL3 and GH3 specimens.

Mix No	S.A m^2^/g	Vt cm^3^/g	Vm cm^3^/g	dpmax (nm)	D50 (nm)	Isotherm type
GL3	5.02	0.063	1.15	105.64	88.12	IV/H3
GH3	18.46	0.190	4.24	59.48	41.25	IV/H3

From a mechanical point of view, the link between microstructure and compressive strength is mainly affected by the presence of large connected pores, coarse capillary pores and microcracks rather than the existence of fine gel pores. The microstructure with large pores has a negative impact on compressive strength. Therefore, the significant shift of the GH3 PSD toward smaller characteristic sizes (dpmax of 59.48 nm and D50 of 41.25 nm) compared to GL3 (dpmax of 105.64 nm and D50 of 88.12 nm) offers a mechanistic explanation that aligns with the observed development in the compressive strength (18.6 MPa for GL3 vs 64.2 MPa for GH3). Pore-size refinement decreases the chance of stress concentration at large defects and enhances the gel network’s load-bearing efficiency.

## Conclusion

This study aims to evaluate the influence of the chemical composition of metakaolin, particularly SiO_2_ and Al_2_O_3_, and their subsequent effect on the amount of Na_2_SiO_3_ required to achieve a specific SiO_2_/Al_2_O_3_ molar ratio on the properties of metakaolin-based geopolymer. The compressive strength was measured and the results were supported by different analysis techniques, such as XRD, TGA/DTG, SEM and BET/BJH models. The following conclusions can be drawn:

- Calcining kaolin at 700 °C for 1 h is enough to produce reactive metakaolin. The Chapelle pozzolanicity values for metakaolin samples are 767 and 1006 mg CaO consumed/g Mk for MkL and MkH, respectively. This indicates that the chemical composition such as Al_2_O_3_ content has significantly influenced the reactivity of metakaolin.
- The results showed that the source of silica in the geopolymer plays a vital role in determining the quality of the final geopolymeric products. The compressive strength was more significantly affected by the amount of soluble SiO_2_ from the activator (Na_2_SiO_3_) than by the total SiO_2_ content in the metakaolin as measured by XRF. This indicates that the reactivity/availability of silica primarily controls gel formation and strength.
- The highest compressive strength values of both metakaolin-based geopolymer prepared from MkH and MkL are obtained at a SiO_2_/Al_2_O_3_ molar ratio of 3.5.
- The geopolymer prepared from MkH has higher compressive strength than those made from MkL. At a SiO_2_/Al_2_O_3_ molar ratio of 3.5, the strength is higher by 245.2%. This highlights the significant impact of the mineralogical composition of metakaolin, even though the prepared geopolymers have the same SiO_2_/Al_2_O_3_ molar ratio.
- Increasing the SiO_2_ content in the metakaolin geopolymeric matrix by adding Na_2_SiO_3_ results in increasing the amount of strength-giving phases, N–A–S–H gel, resulting in a compact microstructure.

## Data Availability

All data generated or analysed during this study are included in this published article.
